# Small Cationic DDA:TDB Liposomes as Protein Vaccine Adjuvants Obviate the Need for TLR Agonists in Inducing Cellular and Humoral Responses

**DOI:** 10.1371/journal.pone.0034255

**Published:** 2012-03-28

**Authors:** Anita Milicic, Randip Kaur, Arturo Reyes-Sandoval, Choon-Kit Tang, Jared Honeycutt, Yvonne Perrie, Adrian V. S. Hill

**Affiliations:** 1 The Jenner Institute, University of Oxford, Oxford, United Kingdom; 2 Aston Pharmacy School, Aston University, Birmingham, United Kingdom; Fundació Institut d'Investigació en Ciències de la Salut Germans Trias i Pujol. Universitat Autònoma de Barcelona. CIBERES, Spain

## Abstract

Most subunit vaccines require adjuvants in order to induce protective immune responses to the targeted pathogen. However, many of the potent immunogenic adjuvants display unacceptable local or systemic reactogenicity. Liposomes are spherical vesicles consisting of single (unilamellar) or multiple (multilamellar) phospholipid bi-layers. The lipid membranes are interleaved with an aqueous buffer, which can be utilised to deliver hydrophilic vaccine components, such as protein antigens or ligands for immune receptors. Liposomes, in particular cationic DDA:TDB vesicles, have been shown in animal models to induce strong humoral responses to the associated antigen without increased reactogenicity, and are currently being tested in Phase I human clinical trials. We explored several modifications of DDA:TDB liposomes - including size, antigen association and addition of TLR agonists – to assess their immunogenic capacity as vaccine adjuvants, using Ovalbumin (OVA) protein as a model protein vaccine. Following triple homologous immunisation, small unilamellar vesicles (SUVs) with no TLR agonists showed a significantly higher capacity for inducing spleen CD8 IFNγ responses against OVA in comparison with the larger multilamellar vesicles (MLVs). Antigen-specific antibody reponses were also higher with SUVs. Addition of the TLR3 and TLR9 agonists significantly increased the adjuvanting capacity of MLVs and OVA-encapsulating dehydration-rehydration vesicles (DRVs), but not of SUVs. Our findings lend further support to the use of liposomes as protein vaccine adjuvants. Importantly, the ability of DDA:TDB SUVs to induce potent CD8 T cell responses without the need for adding immunostimulators would avoid the potential safety risks associated with the clinical use of TLR agonists in vaccines adjuvanted with liposomes.

## Introduction

Majority of vaccines currently in development belong to the category of subunit vaccines, consisting of recombinant or purified pathogen-specific proteins, or encoded (DNA) antigens that will be expressed and presented *in vivo*. Administered alone subunit vaccines have low efficacy in activating the immune system and require the addition of adjuvants in order to induce a measurable immune response to the antigen (Ag), through the activation of the innate, and subsequently the adaptive, immune system. Ideally, the adjuvant should be able to improve Ag uptake by antigen presenting cells (APCs) and induce an Ag-specific immune response while eliciting minimal toxicity. Liposomes are a type of adjuvant that can potentially satisfy all these criteria.

Liposomes are lipid-bilayer vesicular structures within which various substances can be entrapped, and delivered *in vivo* in a discrete and safe manner, protected from degradation. Administration of therapeutic agents inside liposomes has been employed over several decades in enzyme replacement therapy [1], [2], intracellular delivery of chelating agents in cases of heavy metal poisoning [3] and treatment of cancer [4]. More recently, liposomes have found application as vaccine adjuvants [5], [6], [7]: the ability to prevent Ag degradation and clearance, coupled with enhancing its uptake by professional APCs, have marked liposomes as useful vehicles for the delivery of a diverse array of vaccine antigens [8], [9], [10].

The choice of the lipid used in the synthesis of liposomes affects their physico-chemical and immunogenic properties, and extensive research using many diverse lipids, in particular phospholipids, has been carried out with the aim of increasing and optimising the adjuvanting effect of liposome-delivered antigens (reviewed in [11], [12]). Phospholipid molecules contain a non-polar region (composed of one or more fatty acid chains, or cholesterol) and a polar region consisting of a phosphate group linked to tertiary or quarternary ammonium salts. The polar region can have a net negative (anionic), neutral or positive (cationic) surface charge, which is one of the main determinants of liposome behaviour and function. More specifically, liposomes incorporating the synthetic amphiphilic cationic lipid compound dimethyldioctadecylammonium (DDA) combined with an immunostimulatory component, trehalose 6,6-dibehenate (TDB), a non- toxic analogue of the mycobacterial cell wall component trehalose 6,6′ dimycolate (TDM), have been shown to strongly enhance cellular and humoral responses against a protein antigen [13]. Adjuvanticity of the cationic DDA:TDB liposomes and sustained protection against disease challenge has been demonstrated in particular with a tuberculosis vaccine candidate [14], [15] and has good potential for application in a range of other diseases [16].

The antigen to be delivered can be either entrapped within the aqueous compartment of the liposomes, incorporated into the lipid bilayer membrane (hydrophobic antigens) or adsorbed to the liposomal surface through covalent or charge-dependent, electrostatic, interaction [17], [18], [19] and past studies have addressed the relative merits of the Ag/liposomal vesicle configuration in enhancing the adjuvant effect of liposomes [20]. More recently, with the advanced recognition of the roles of innate pathogen receptors in adaptive immunity, researchers have been exploring the potential for enhancing immunogenicity of cationic liposomes through addition of Toll-Like Receptor (TLR) agonists [21], [22], [23]. In turn, liposome encapsulation of CpG oligonucleotides has been shown to enhance and prolong innate system stimulation and significantly improved the CpG-induced immune protection against *Listeria* [24], [25].

TLRs signal through two main intracellular pathways: MyD88-dependent (TLRs 2, 4, 5 and 9) and MyD88–independent (TLRs 3 and 4) [26]. Signalling through both pathways simultaneously was shown to have a synergistic effect on their ability to induce pro-inflammatory cytokine production in mice [27], whereas multiple agonists of the same pathway can be mutually antagonistic [28]. Unlike the cell surface-bound members of the TLR family, several of these receptors (TLRs 3, 7, 8 and 9) reside within the endosomal cellular compartment [29], [30] and thus encounter internalised antigens. It has been shown that endosomally located TLRs can enhance Ag presentation by DCs through the MHC class I pathway (cross-presentation), resulting in increased murine CD8 T cell responses [31], [32].

Here we describe a study conducted to further examine the adjuvant properties of cationic liposome/TLR agonist complexes in inducing humoral and cellular responses to a protein Ag, and to extend previous observations by investigating the effect of liposome size and lamellarity. We focused on DDA:TDB cationic liposomes combined with TLR3 and TLR9 agonists (poly I:C and CpG ODN M362, respectively) and used whole Ovalbumin protein (OVA) as a model protein vaccine. As mentioned above, TLRs 3 and 9 are both endosomally located, although they differ in their requirement for MyD88 in the downstream signalling [28]. Poly I:C and CpG ODN were delivered either entrapped inside the liposomes or adsorbed to the liposomal surface, in order to assess whether encapsulating these TLR agonists within liposomes is more likely to activate their endosomally located receptors and, in particular, enhance CD8 T cell responses to OVA protein.

## Results

### Liposome formulations

We investigated three types of DDA:TDB liposomes of different size and lamellarity. Multilamellar vesicles (MLVs) are around 500 nm in diameter and consist of several concentric lipid bi-layers. Dehydration-rehydration vesicles (DRVs) are lipid bi-layer membrane vesicles which encapsulate the antigen and/or TLR agonist. Small unilamellar vesicles (SUVs) are single membrane vesicles, around 60 to 120 nm in diameter. The diameter of both MLVs and SUVs increased with the addition of protein Ag. Liposomes were formulated with whole OVA protein either attached to the liposomal surface through electrostatic forces (MLV, DRV and SUV formulations) or entrapped within the liposomes (DRV formulations). Some of the DDA:TDB/OVA formulations were further combined with TLR agonists poly I:C (pIC) and/or CpG, which were again either surface-adsorbed or entrapped. Analysis of the proportion of the OVA protein retained within the different liposomal formulations over time demonstrated good stability in Tris buffer. Under simulated *in vivo* conditions (Tris buffer supplemented with 50% FCS and incubated at 37°C), although there was an increased release of OVA, over 50% of the antigen was still associated with the liposomes after 96 h of incubation (Figure S1).

### Liposome characterisation

**Figure 1:**
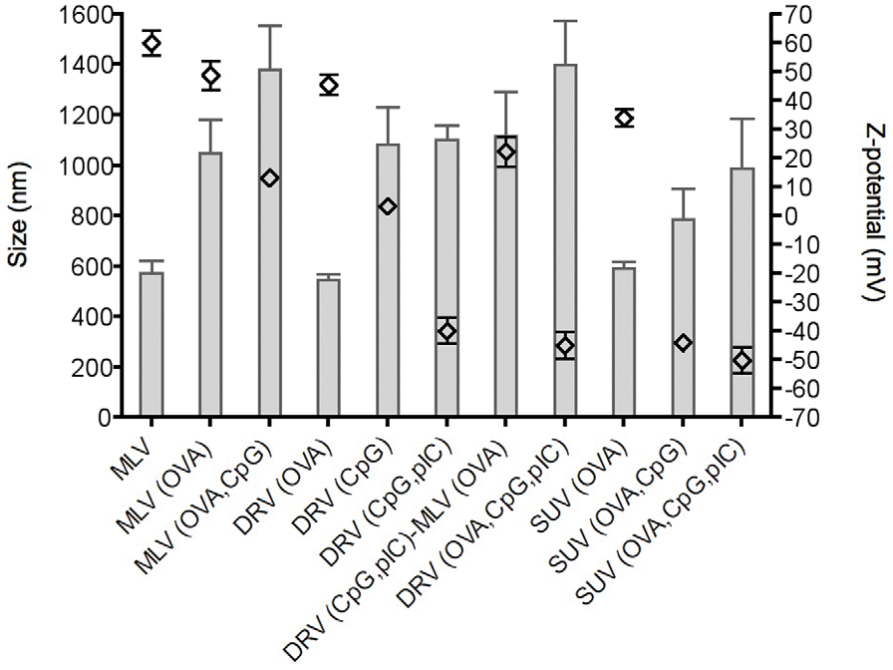
Size distribution (bars) and Z-potential (points) of DDA:TDB liposomal formulations with OVA±pIC/CpG.

Physico-chemical characterisation of all liposomal formulations was carried out by measuring the size, polydispersity, Zeta (Z)-potential and the proportion of OVA protein incorporated in the formulations (Table 1 and Figure 1). Addition or incorporation of the negatively charged OVA protein and nucleic acid-based pIC and CpG into the cationic liposomes was found to affect the particle size and zeta potential. Empty DDA:TDB MLV liposomes to which soluble OVA was added were 667.2±72.6 nm in diameter, with a positive charge (Z-potential = 46.26±3.7 mV). Adsorption of OVA resulted in a size increase to 1047.1±135.8 nm, due to aggregation of vesicles promoted by antigen interactions, with a negligible rise in Z-potential. Further addition of CpG to the liposome surface resulted in a non-significant size increase and a drop in Z-potential to 13.2±5.3.

**Table 1:** Liposome specifications of the DDA:TDB formulations containing OVA and TLR agonists.

Formulation	Size (nm) ± SD	Z-potential (mV) ± SD	Polydispersity Index (PDI)	Proportion OVA incorporated(% of total dose)
MLV+sOVA	667.2±72.6	46.26±3.7	0.321±0.02	86%
MLV(OVA)	1047±135.8	48.7±12.1	0.367±0.01	95%
MLV(OVA,CpG)	1378.6±176	13.2±5.3	0.463±0.02	44%
DRV(OVA)	546±23.7	45.4±8.6	0.353±0.01	93%
DRV(CpG)+sOVA	1234±26.7	−3.7±5.6	0.336±0.01	74%
DRV(pIC,CpG)+sOVA	1336.2±35.8	−40.2±6.7	0.410±0.01	80%
DRV(pIC,CpG)+MLV(OVA)	1116.9±176	22.26±12.37	0.359±0.08	87%
DRV(OVA,pIC,CpG)	1399.2±172.5	−44.9±11.1	0.355±0.01	85%
SUV(OVA)	591.9±25.7	33.98±7.2	0.415±0.03	85%
SUV(OVA,CpG)	785.8±123.1	−43.99±3.7	0.403±0.01	56%
SUV(OVA,pIC,CpG)	985.8±200	−50.12±11	0.442±0.01	60%

Liposomes encapsulating OVA, DRV(OVA), were significantly smaller than the vesicles prepared with surface-complexed OVA (546±23.7 vs 1047.1±135.8 nm). Entrapment of CpG alone increased the liposome size to 1234±26.7 nm and reduced the Z-potential to below neutral (−3.7±5.6 mV), suggesting a reconfiguration of the system in comparison to DRV(OVA). Entrapment of pIC in combination with CpG resulted in vesicles of a similar size (1336.2±35.8 nm) with a negative zeta potential (−40.2±6.7 mV), indicating that some of the material may also be electrostatically adsorbed to the surface of the DRV as well as entrapped, thereby masking the cationic nature of the liposome surface. Entrapment of all three components OVA, CpG and pIC resulted in no significant change in zeta potential and led to a further increase in size to 1399.2±172.5 nm.

Empty DDA:TDB SUV liposomes have an average size of 67.8±12.8 nm. Surface-adsorption of OVA increased their size to 591.9±25.7 nm, and adding CpG alone or with pIC resulted in even larger liposomes (785.8±123.1 nm and 985.8±200 nm, respectively). The addition of the TLR agonists also inverted the Z-potential from 33.98±7.2 mV to −43.99±3.7 mV and −50.12±11 mV, respectively.

### SUVs induce strong CD8 T cell and antibody responses to OVA

**Figure 2:**
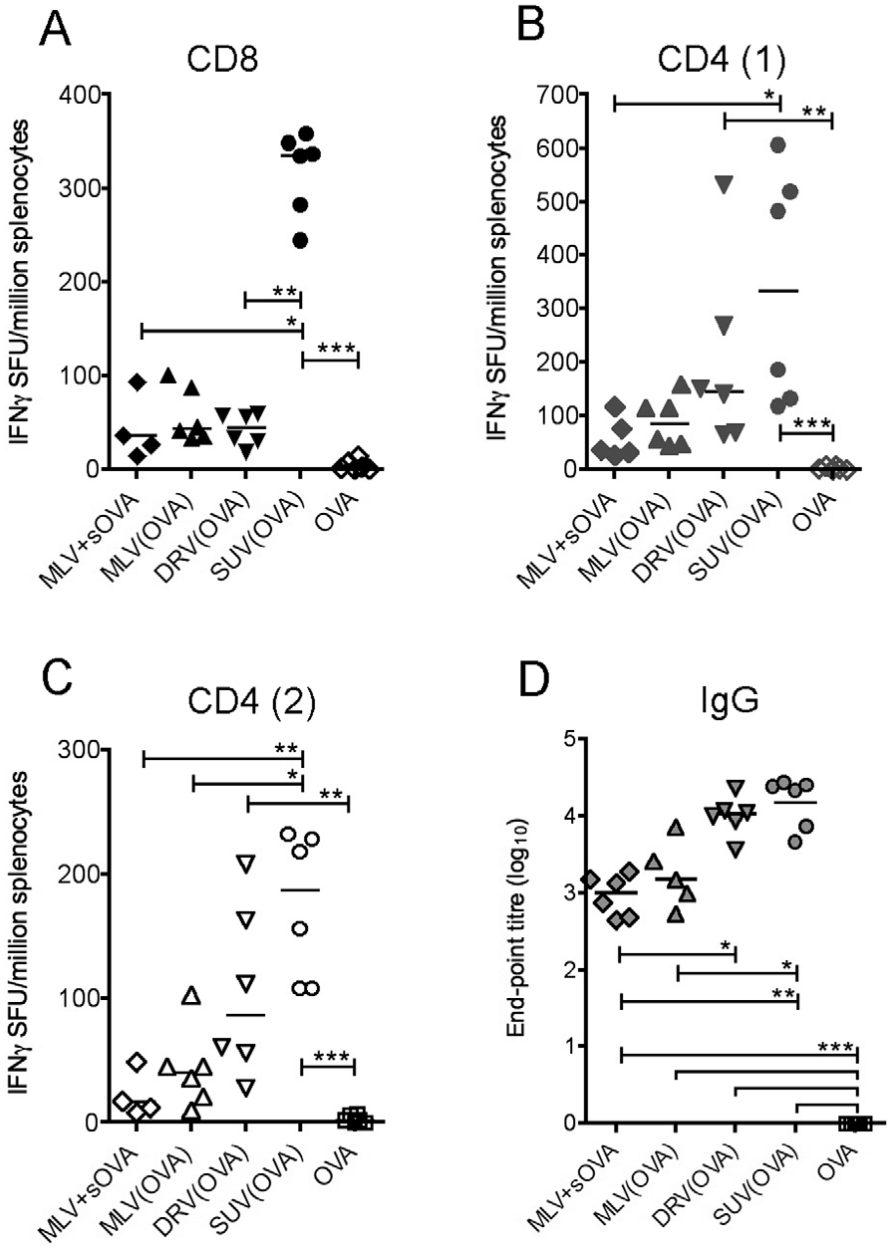
Cellular and humoral responses following different liposome + OVA formulations. The DDA:TDB+OVA formulations indicated along the X-axis were given to C57/BL6 mice as three homologous injections, two weeks apart. Splenocytes were isolated at the final timepoint (14 weeks) and responses to a CD8 OVA epitope (A) and two CD4 epitopes (B, C) were assessed by *ex vivo* IFNγ ELISpot. D) Total IgG antibody responses to whole OVA protein at the final time-point, as measured by an end-point titre ELISA. *p<0.05, ** p<0.01.

We first investigated the adjuvant potential of DDA:TDB liposomes formulated with OVA protein alone, with no added TLR agonists (Figure 2). One mouse-dose of each liposome formulation contained 250 µg DDA and 50 µg TDB; one dose of OVA was 20 µg. We tested SUVs and MLVs with surface-adsorbed OVA protein as well as DRVs with OVA entrapped within the liposomes, in a regime consisting of three homologous injections of each formulation intramuscularly, two weeks apart. Animals were followed-up for 10 weeks post last injection and total IgG responses in the serum measured two weeks after every vaccination and at two further time-points (10 and 14 weeks from first immunisation). At the final time-point (14 weeks) the animals were sacrificed and spleen CD4 and CD8 T cell IFNγ responses assessed.

Adsorbing OVA to SUVs (SUV(OVA)) strongly enhanced the spleen CD8 IFNγ responses (p<0.01) (Figure 2A). MLVs with surface-adsorbed OVA (MLV(OVA)) and DRVs encapsulating the protein (DRV(OVA)) did not significantly increase CD8 (Figure 2A) nor CD4 (Figure 2B, 2C) responses as compared to MLVs combined with OVA in solution (MLV+sOVA). A triple immunisation with OVA protein alone did not induce any CD4 or CD8 responses. The CD4 IFNγ responses in the spleen were also significantly higher following vaccination with SUV(OVA) in comparison to MLV+sOVA (p<0.05). Median responses after DRV(OVA) vaccination were higher for both CD8 and CD4 splenocytes as compared with MLV(OVA) liposomes, but did not reach statistical significance (Figure 2A, 2B and 2C).

Total IgG titres against whole OVA protein were lowest in the animals vaccinated with MLV+sOVA and similar to MLVs with surface adsorbed OVA. At the peak timepoint (2 weeks post last injection) end-point titres in the DRV(OVA) and SUV(OVA) groups were up to 100-fold higher than those receiving the MLV formulations. The significantly higher titres in these two groups persisted until the final timepoint (p<0.01 for SUV(OVA) and p<0.05 for DRV(OVA) when compared to MLV+sOVA, Figure 2D).

### Addition of TLR3 and TLR9 agonists significantly increases CD8 T cell immunogenicity of MLV(OVA) and DRV(OVA), but not SUV(OVA)

**Figure 3:**
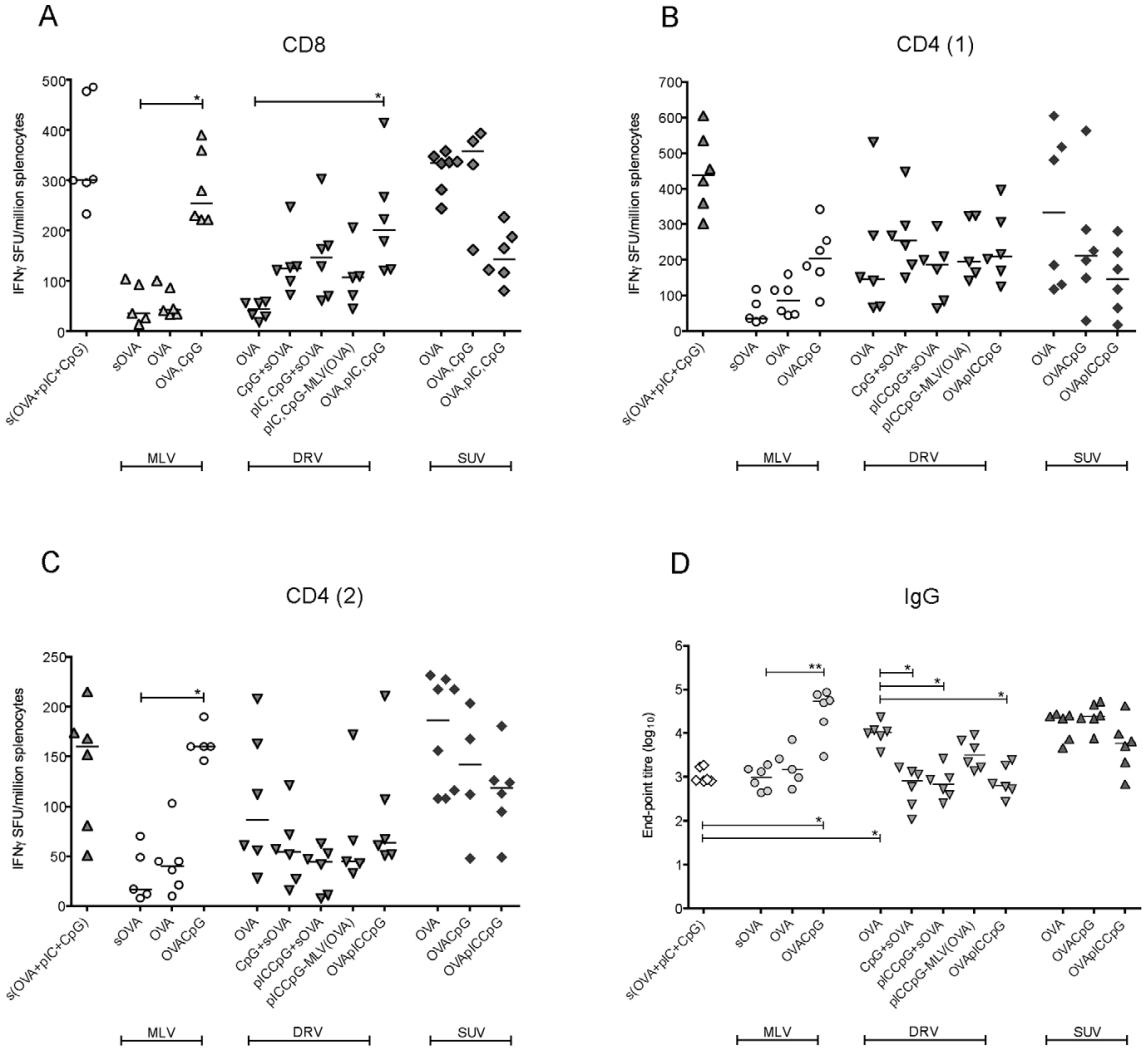
Cellular and humoral responses to DDA:TDB liposomal formulations with OVA±TLR3 and TLR9 agonists. Following three homologous injections of each DDA:TDB liposomal formulation, T cell and antibody responses were assessed at the terminal time-point (14 weeks). Mouse splenocytes were isolated and T cell responses to an OVA CD8 epitope (A) and two CD4 epitopes (B and C) measured by IFNγ ELISpot. D) Total IgG titre in the serum at the terminal timepoint, assessed by end-point ELISA. *p<0.05, **p<0.01.

We then tested the immunostimulatory effect of adding pIC and CpG ODN to the liposome/OVA formulations, in an identical vaccination regimen of three homologous injections two weeks apart (Figure 3). The doses of TLR agonists administered per immunisation were 20 µg CpG and 50 µg pIC.

Adding CpG to MLV(OVA) increased the CD8^+^IFNγ^+^ responses (Mann-Whitney unpaired t-test, p = 0.002) to levels comparable to those observed after vaccination with OVA, pIC and CpG in solution (sOVA+pIC+GpC), despite the reduced proportion of the incorporated OVA antigen (44%, Table 1). Entrapping poly I:C and CpG within DDA:TDB liposomes (DRV(OVA, pIC, CpG)) also resulted in significantly enhanced CD8 responses (Welch's unpaired t-test for unequal variances, p = 0.0028) compared to DRV(OVA), although marginally lower than sOVA+pIC+GpC. Adsorbing CpG along with OVA on SUVs preserved the high CD8 response observed with SUV(OVA) alone, with both medians higher than that of s(OVA+pIC+GpC). Conversely, the addition of pIC resulted in a decrease of the CD8 response (p<0.05) (Figure 3A).

For one of the two CD4 epitopes assessed (Figure 3B), addition of CpG to MLV(OVA) resulted in a significantly higher CD4 response (p = 0.002), equal to that induced by s(OVA+pIC+GpC). The CD4 responses measured after vaccination with DRVs containing OVA and TLR agonists showed relatively low but comparable median values. Among the SUV liposomal formulations tested, analogous to the observed CD8 response, the highest proportion of CD4^+^IFNγ^+^ cells were induced with the formulation lacking the TLR agonists, the addition of which resulted in a non-significant reduction of the CD4 response.

Antibody titres at the final time-point (14 weeks) showed a similar trend to the T cell responses with respect to the addition of pIC and CpG to the liposome/OVA formulations (Figure 3D). Adsorbing CpG to MLV(OVA) resulted in significantly higher titres as compared to MLV+sOVA (p<0.009) and reached highest median titre of all of the tested formulations. This formulation also induced a stronger humoral response than the non-liposomal combination of the three components (p<0.05). In contrast to CD8 responses, DRV(OVA) was significantly more potent in inducing IgG titres compared to the pIC and CpG-containing DRV formulations (p<0.05). SUV liposomes also produced high IgG responses, although a slight decrease was detected when both CpG and pIC were added to SUV(OVA). It should be noted that several formulations, both with and without TLR agonists, resulted in significantly higher titres than s(OVA+pIC+CpG). Analysis of the relative proportions of IgG subclasses induced in peripheral blood demonstrated that all of the formulations resulted in IgG2a/IgG1 ratio of around 1, indicating no preferential Th1 or Th2 antibody response (data not shown).

### Unlike DDA:TDB, neutral (DSPC/TDB) liposomes do not interfere with the immunogenicity of viral vectored vaccines

As mentioned previously, protein vaccines are poorly immunogenic. In contrast, potent immune responses, humoral, and in particular cellular, can be induced if the antigen is delivered encoded within a viral vector. To investigate whether liposomes as adjuvants would interfere with immunogenicity of such vectors, we tested cationic (DDA:TDB) and neutral (DSPC/TDB) SUVs and MLVs in combination with two non-replicating viral vectors, adenovirus (Ad) and Modified Vaccinia Ankara (MVA).

**Figure 4:**
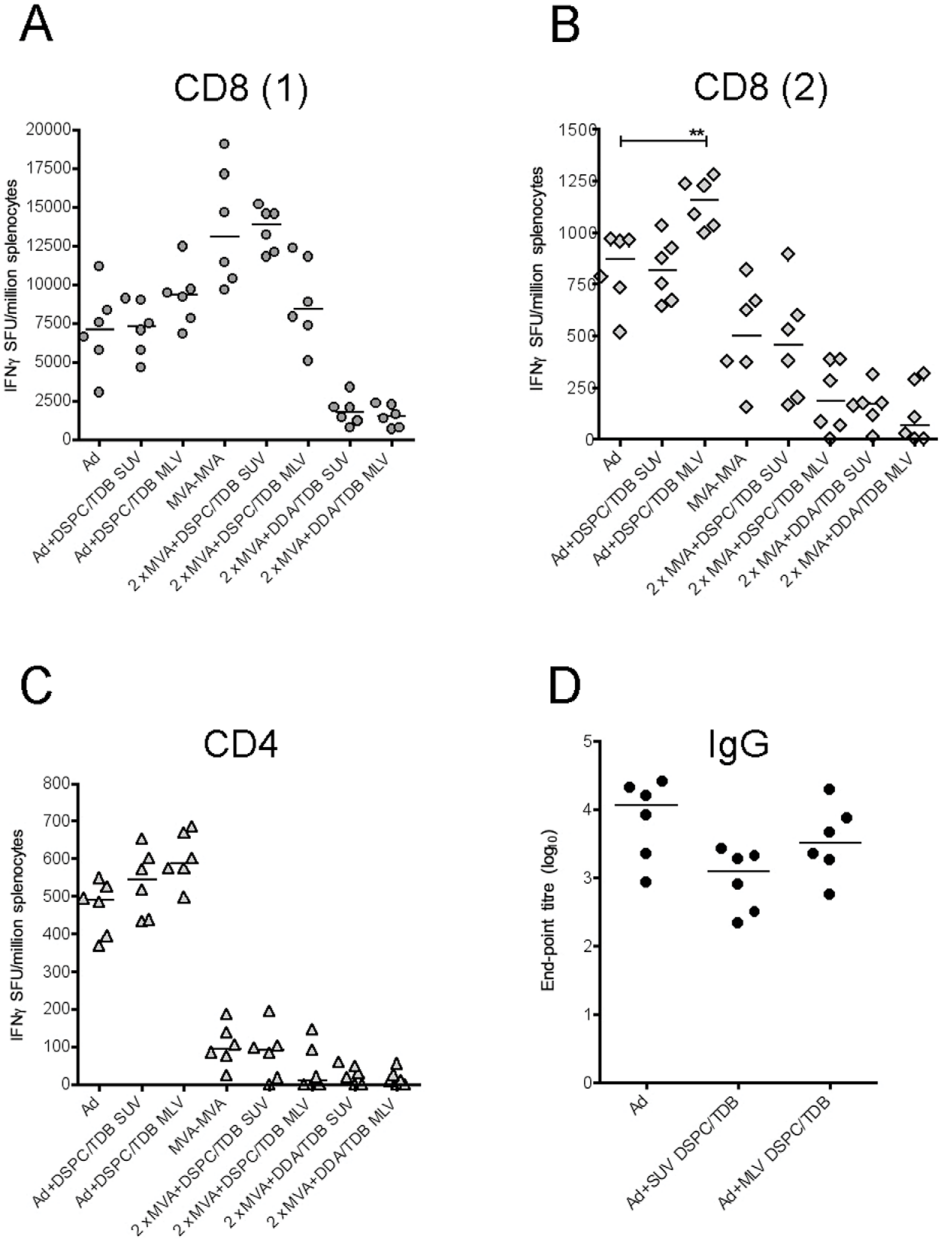
Cellular and humoral responses to liposome-adjuvanted viral vectored vaccines. T cell and antibody responses were assessed in Balb/c mice at peak time-points for Adenovirus (Ad) and Modified Vaccinia Ankara (MVA) viral vectored vaccines expressing TIPeGFP, i.e. two weeks after a single injection of Ad and one week after two injections of MVA, combined with cationic DDA:TDB or neutral DSPC/TDB liposomes. Spleen T cell responses to two CD8 (A, B) and one CD4 epitope (C), contained within the insert of the viral constructs, were measured using IFNγ ELISpot. D) Total IgG titre in the serum of mice immunised with the Ad+liposome formulations at the peak time-point. **p<0.01.

We assessed immune responses following a single injection of Ad or two injections of MVA (prime-boost, 1 week apart), both encoding TIPeGFP - a string of CD4 and CD8 T cell epitopes and GFP protein as a model Ag for measuring antibody responses - with or without liposomes (Figure 4). We found that cationic liposomes, both SUVs and MLVs, completely abrogated cellular and humoral immune responses induced by the adenoviral construct Ad-TIPeGFP, and to a large degree the T cell responses following MVA-TIPeGFP vaccination. This was anticipated, as the surface of the Ad virus is strongly negatively charged and cationic DDA:TDB liposomes would have occluded the CAR receptor and prevented cell entry of the Ad virus. The majority of the MVA particles are enveloped by the cell membrane, which is also negatively charged, and would be similarly likely to be coated by cationic liposomes that would interfere with cell entry.

We therefore tested neutral distearoyl-glycero-3-phosphocholin/TDB (DSPC/TDB) SUV and MLV liposomes in combination with the same viral vectors. Due to the lack of electric charge, these liposomes form larger vesicles with more lipid bilayers in comparison to cationic: neutral SUVs had a diameter of 135±20.6 nm and MLVs 1560±240 nm. The DSPC/TDB liposomes had no significant effect on the cellular immunogenicity induced by MVA in the spleen, although a decreasing trend was observed with MLVs for both CD4 and CD8 epitopes when compared to MVA-only vaccination (Figure 4A, 4B, 4C). In contrast, with Ad vector-delivered antigen DSPC/TDB MLVs resulted in a slightly higher CD4 and CD8 responses that reached statistical significance for one of the CD8 epitopes tested (Figure 4B).

No detectable antibody responses against the OVA antigen were induced following two injections of the MVA vectored vaccine, either with or without liposomes. Humoral responses to the Ad-vectored vaccine were not significantly affected by the DSPC/TDB liposomes, although a weak decreasing trend was observed with the SUVs (Figure 4D).

## Discussion

We show here that cationic DDA:TDB liposomes of same chemical composition but different size and lamellarity differ in their ability to induce humoral and cellular immunity when combined with a protein antigen. Small unilamellar liposomes (∼600 nm in diameter when combined with protein Ag) were able to induce significantly higher cellular and humoral adaptive immune responses than multilamellar vesicles with a two-fold larger diameter. The antibody titres measured with SUV(OVA) were higher than those reported previously following the same vaccination regime with several potent adjuvants, including Freund's, ISA 720 and aluminium-based adjuvants [33]. Antigen-specific CD8 T cells in particular were shown to be strongly induced by DDA:TDB SUVs, despite a small reduction in the amount of OVA incorporated into these liposomes compared to MLVs (85% vs 95% of total dose of the OVA protein). This was an unexpected finding, considering that the size difference between these two formulations is only 2-fold, and that the multilamellar vesicles contain a higher proportion of the immunostimulatory component TDB than the single lipid-bilayer SUVs. Without the adsorbed Ag, SUVs and MLVs differ in diameter by around 10-fold and it is possible that a difference in the endocytic pathways involved in the internalisation of the two types of liposomes is affecting Ag presentation and the ensuing immune response [34]. A recent study explored the effect of the size of cationic liposomes on the T- and B cell responses and also observed preferential phagocytosis by macrophages and higher splenic IFNγ production with smaller (∼500 nm) versus larger (>2 µm) liposomes, although, interestingly, there was no enhancement of humoral immunogenicity [35].

The primary aim of this work was to investigate the impact on antigen-specific T cell and antibody responses of delivering the antigen entrapped inside or adsorbed to the surface of the liposomes. Similarly, in light of a previous report showing that endocytosed and subsequently endosomally presented CpG ODN can enhance cross-presentation to CD8 cells by Ag-presenting cells [31], we sought to investigate the effect of delivering TLR agonists CpG and poly I:C, either encapsulated within liposomes or attached to the liposomal surface, on Ag-specific CTL responses in mice.

With respect to Ag location, comparing MLV and DRV liposome formulations, we found that slightly higher antibody titres and CD4 T cell responses, can be achieved by delivering the Ag encapsulated within the liposomes, as compared to a surface-adsorbed Ag. Although the trend observed in our system was not statistically significant, it lends support to an earlier study that showed an enhancement in Ab production when BSA protein was delivered inside phosphatydylcholine/cholesterol liposomal vesicles [20] and suggests that Ag entrapment could lead to better B cell recognition through augmenting CD4 T cell help. A recent study comparing immune responses to the entrapped versus adsorbed tuberculosis antigen Ag85-ESAT-6 in Balb/c mice, however, found that both DDA:TDB MLV and DRV formulations appeared to result in similar IgG titres [36].

Cationic DDA:TDB liposomes have been considered unable to elicit potent CD8 responses without the addition of a TLR agonist [15] and our results support previous reports that TLR3 and TLR9 agonists can help generate CD4 and CD8 T cell responses to a liposome delivered Ag *in vivo* [22]. However, the added TLR agonists were only able to enhance immune responses when combined, either entrapped or surface adsorbed, with larger liposomes (MLVs or DRVs). In contrast, the smaller SUV liposomes induced strong CD8 IFNγ production in the absence of TLR agonists, the addition of which even led to a decrease (non-significant) of the assayed immune responses. The cause of this decrease in immunogenicity, which was mainly evident in the T cell responses, is not clear, although we noted two side-effects arising from adding TLR agonists to SUV liposomes. One was an increase in the liposomal diameter to nearly twice that of SUVs containing OVA only, and the other a reduction in the amount of liposome-entrapped OVA protein with the inclusion of CpG and poly I:C by around a third. Either or both of these could have led to the observed reduction in the CD4 and CD8 responses in this group. Another recent study looking at encapsulation of OVA and TLR agonists PAM_3_CSK and CpG within cationic liposomes also observed a 25% reduction in the entrapped OVA when the TLR agonists were added, as well as no increase in the total IgG titres in the animals receiving this formulation, thus supporting our findings [23].

A previous study by Tanaka et al. [37] investigated the role of saturated and non-saturated fatty acid liposomes in Ag targeting to the class I or class II processing pathways *in vitro*. They found that liposomes composed of stearoyl acid (saturated fatty acid with 18 C chain) were more effective in targeting the antigen to the MHC class II processing pathway, whilst oleoyl (18 C fatty acid with a single double bond) liposomes were successfully targeting OVA to both class I and II processing. Our study employed liposomes composed of DDA, a saturated fatty acid containing 18 C residues and hence equivalent to stearoyl acid. Our observation of a significant enhancement of both CD4 and CD8 Ag-specific responses with SUV liposomes as compared to MLVs suggests that the liposomal size and lamellarity, rather than the number of double bonds in the fatty acid chain, could be a more significant determinant in targeting the antigen for MHC class I or II presentation. Similarly, using small unilamellar liposomes appears to circumvent the need for a pH-sensitive liposome delivery system, which has been shown to induce CTL response against the protein antigen [38].

We also assessed the ability of liposomes to enhance immunogenicity of viral vectored vaccines, which are intrinsically immunogenic and difficult to adjuvant further. As predicted, cationic liposomes completely abrogated the immunogenicity of the Adenovirus vectored vaccine, probably due to the opposing surface charges between DDA:TDB and the negatively charged Ad virus, which would lead to liposome coating of the virus and prevent cell entry. Similar effect was observed with the MVA vectored vaccine (where most particles would be enveloped by a cell membrane and hence also negatively charged) and cationic liposomes. Non-charged DSPC/TDB liposomes did not have the neutralising effect of the DDA:TDB vesicles, and were able to enhance both CD4 and CD8 T cell responses induced by the Ad vector. MLVs in particular were more potent than SUVs in adjuvanting the Ad vector. It has been shown that Adenovirus can enter the cell through macropinocytosis [39], [40] and the liposomes administered with the Ad vector are likely to have been taken up by the cells through the same mechanism [41]. This could explain the observed increase in CD8 T cell responses to the Ad vector delivered antigen, as macropinocytosis has been implicated in cross-presentation by DCs in vivo [42], [43].

In summary, our study demonstrates that addition of TLR agonists CpG and poly I:C to cationic DDA:TDB liposomes formulated with protein Ag can enhance the immunogenicity of MLV but not SUV or DRV liposomes, as measured by both pro-inflammatory cellular (CD4 and CD8) and IgG antibody responses. This finding was partially due to the potent adjuvanting effect of the small unilamellar DDA:TDB vesicles alone, which was equivalent to, or higher than, that measured with the protein Ag with TLR agonists in solution. The observations presented here could be of use when choosing liposomes as protein antigen delivery vehicles and adjuvants, as they could circumvent the need for inclusion of TLR agonists, and the potential clinical safety-related issues associated with TLR agonists, in liposome-adjuvanted vaccines.

## Materials and Methods

### Preparation of liposomes

MLVs were prepared using the previously described lipid-film hydration method (Bangham et al 1965). Briefly, weighed amounts of DDA (or DSPC) and TDB were dissolved in chloroform/methanol (9∶1 by volume) and the organic solvent was removed by rotary evaporation, followed by flushing with N_2_ to form a thin lipid film on the bottom of a round-bottomed flask. The lipid film was hydrated in 10 mM Tris-buffer at pH 7.4 by heating for 20 min at 60°C.

To generate SUVs, the MLV were disrupted using sonic energy to fracture large liposomes into smaller structures (<100 nm). The tip of a sonicator probe (Soniprep 150) was placed onto the liposome surface of the MLV mixture, transforming the milky MLV suspension into a clear SUV suspension.

For the preparation of DDA:TDB DRVs, SUVs were stirred in the presence or absence of OVA, CpG and poly I:C, frozen at −70°C, and freeze dried overnight (−40°C, vacuum to 40 mbar). Controlled rehydration of the dried powder, leading to the formation of antigen-containing DRVs, was achieved by addition of distilled water in aliquots equalling 10% of the final volume (which was standardised at 1 mL), with vortexing and 30 min incubation at room temperature following addition of each aliquot [44]. DRV preparations were then centrifuged twice at 45,000 rpm for 20 min to remove non-entrapped components and resuspended in PBS to the appropriate volume. To determine antigen adsorption/entrapment, OVA (grade VII, Sigma, UK) was radio-labelled with I^125^ and liposome formulations were prepared as described above.

### Determination of vesicle size and zeta potential

Samples were diluted in 10 mM Tris-buffer at pH 7.4 to achieve optimal vesicle concentration. Vesicle size and zeta-potential were determined using photon correlation spectroscopy (PCS), at 25°C, using ZetaPlus (Brookhaven Instrument Corporation, USA).

### Mice and immunizations

Ethics Statement: All procedures were performed under the UK Home Office personal project licence PPL 30/2414, and approved by the University of Oxford Animal Care and Ethical Review Committee, in accordance with the terms of the UK Animals (Scientific Procedures) Act Project Licence.

Female BALB/c (H-2^d^) or C57BL/6 (H-2^b^) mice 6–8 weeks of age (Harlan Laboratories, Oxfordshire, UK) were anesthetized with Isofluorane (Isoflo, Abbot Animal Health, UK) prior to the immunizations. All immunisations were administered intramuscularly (i.m.) into the gastrocnemius muscle by delivering 25 µl per limb (50 µl per mouse). Three injections of each OVA/liposome formulation were given at two weeks intervals. Doses of each component per injection per mouse were as follows: OVA 20 µg, DDA:DSPC 250 µg, TDB 50 µg, CpG 20 µg and poly I:C 50 µg.

AdHu5 and MVA viral vectors containing an insert consisting of TIP (described in [45]) and Green Fluorescent Protein (GFP), named TIPeGFP, were administered at 10^6^ pfu/mouse (MVA TIPeGFP) and 10^8^ iu/mouse (Ad TIPeGFP). In all animals the immune responses were assayed two weeks after each immunization.

### ELISA

Total IgG ELISA was carried out as described previously [46]. Briefly, whole OVA or GFP protein was adsorbed to 96-well Nunc-Immuno Maxisorp plates (Fisher Scientific, Wohlen, Germany) at 2 µg/mL final concentration. Mouse sera were typically diluted to 1∶100, added to wells in duplicate and further serially diluted. Bound antibodies were detected using alkaline phosphatase-conjugated goat anti-mouse total IgG (Sigma) followed by incubation with extravidin alkaline phosphatase conjugate (Sigma, UK). Plates were developed by adding *p*- nitrophenylphosphate substrate (Sigma, UK) and optical density read at 405 nm (OD_405_) once the colour started developing. A positive serum sample of known OD was added as control for each assay. Endpoint titers were taken as the x-axis intercept of the dilution curve at absorbance value 3 standard deviations greater than the OD_405_ for naïve mouse serum (typical cut-off OD_405_ for positive sera = 0.15).

### Ex-vivo IFNγ ELISPOT

The number of IFNγ secreting antigen-specific T cells in fresh splenocyte preparations was determined using the standard IFNγ ELISpot assay. In brief, 96-well MAIP ELISPOT plates (Millipore, UK) were coated with anti-mouse-IFNγ mAb (Mabtech, UK) at 5 µg/mL in carbonate-bicarbonate buffer. Plates were blocked with DMEM (Sigma Aldrich, UK) containing 10% FBS (Biosera Ltd, UK), 2 mM L-glutamine, 100 U/mL penicillin and 100 µg/mL streptomycin sulphate (all from Invitrogen, UK) for 1 h at 37°C. Following the removal of erythrocytes using ACK Lysis buffer, splenocytes from immunised mice were added in duplicate to the coated wells at 500,000, 250,000 and 125,000 cells per well. Peptides corresponding to known OVA CD8 and CD4 T cell epitopes SIINFEKL (OVA_257–264_), TEWTSSNVMEERKIKV (OVA_265–280_) and ISQAVHAAHAEINEAGR (OVA_323–339_), or epitopes contained within the viral vectored insert TIPeGFP: the H2-K^d^-restricted SYIPSAEKI (from P. berghei), HYLSTQSAL (from GFP) and TFLTSELPGWLQANRHVKPT (tuberculosis antigen 85A P15 epitope) were added to the test wells at the final concentration of 1–5 µg/mL. Following overnight incubation, cells were discarded and plates incubated with biotinylated anti-mouse-IFNγ mAb (Mabtech, UK), followed by a streptavidin alkaline phosphatase polymer (Mabtech, UK). Spots were developed by addition of colour development reagent (Alkaline Peroxidase conjugate substrate kit, BioRad, UK) and counted using ELISPOT software (Autoimmun Diagnostika, Germany). Background responses in non-peptide stimulated wells were subtracted from the test wells. Results are expressed as spot forming cells (SFC) per million cells.

### Statistical analysis

All statistical analyses were done using the statistical software integral to the GraphPad Prism software (GraphPad Software Inc., USA). One-way ANOVA analysis was used for the determination of statistical significance, unless otherwise specified.

## Supporting Information

Figure S1 **OVA retention profile for the liposomal OVA/TLR formulations.** The proportion of OVA retained in (A) MLV, (B) DRV and (C) SUV liposomal formulations, either stored in Tris buffer or in Tris supplemented with 50% FCS at 37°C (simulated *in vivo* conditions). Using I^125^-labelled OVA, aliquots of each formulation were incubated in a shaking water bath at 37°C for 96 h. At the indicated time intervals, samples were centrifuged twice and OVA release was calculated as a percentage of the recovered radioactivity. Results represent Mean ± SD of triplicate experiments.
(TIF)
