# Supplementary figures and images for: Small Cationic DDA:TDB Liposomes as Protein Vaccine Adjuvants Obviate the Need for TLR Agonists in Inducing Cellular and Humoral Responses
Source: PLoS One. 2012 Mar 28;7(3):e34255. doi: 10.1371/journal.pone.0034255 (PMC3314611; doi:10.1371/journal.pone.0034255)

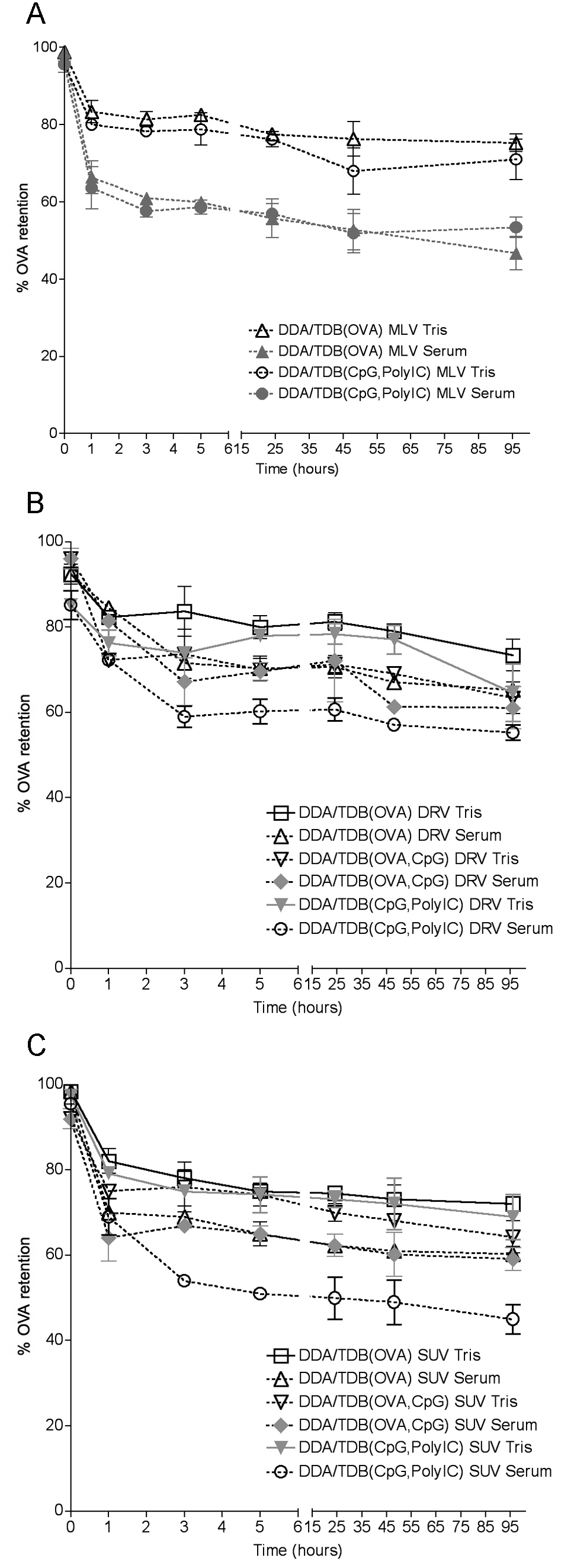

Supplement: Figure S1 — OVA retention profile for the liposomal OVA/TLR formulations. The proportion of OVA retained in (A) MLV, (B) DRV and (C) SUV liposomal formulations, either stored in Tris buffer or in Tris supplemented with 50% FCS at 37°C (simulated in vivo conditions). Using I125-labelled OVA, aliquots of each formulation were incubated in a shaking water bath at 37°C for 96 h. At the indicated time intervals, samples were centrifuged twice and OVA release was calculated as a percentage of the recovered radioactivity. Results represent Mean ± SD of triplicate experiments. (TIF) [file pone.0034255.s001.tif]
